# Intracranial Biodegradable Silica-Based Nimodipine Drug Release Implant for Treating Vasospasm in Subarachnoid Hemorrhage in an Experimental Healthy Pig and Dog Model

**DOI:** 10.1155/2015/715752

**Published:** 2015-01-22

**Authors:** Janne Koskimäki, Miikka Tarkia, Tuula Ahtola-Sätilä, Lasse Saloranta, Outi Simola, Ari-Pekka Forsback, Aki Laakso, Janek Frantzén

**Affiliations:** ^1^Faculty of Medicine, University of Turku, P.O. Box 52, Kiinamyllynkatu 4-8, 20520 Turku, Finland; ^2^Department of Neurosurgery, Helsinki University Central Hospital, P.O. Box 266, Topeliuksenkatu 5, 00029 Helsinki, Finland; ^3^Turku PET Centre, University of Turku and Turku University Hospital, P.O. Box 52, Kiinamyllynkatu 4-8, 20521 Turku, Finland; ^4^Chemistry and Safety Sciences, R&D, Orion Corporation, Orion Pharma, P.O. Box 65, Orionintie 1A, 02101 Espoo, Finland; ^5^Delsitech Ltd., Itäinen Pitkäkatu 4B, 20520 Turku, Finland; ^6^Clinical Neurosciences, Department of Neurosurgery, Turku University Hospital, P.O. Box 52, Hämeentie 11, 20521 Turku, Finland

## Abstract

Nimodipine is a widely used medication for treating delayed cerebral ischemia (DCI) after subarachnoid hemorrhage. When administrated orally or intravenously, systemic hypotension is an undesirable side effect. Intracranial subarachnoid delivery of nimodipine during aneurysm clipping may be more efficient way of preventing vasospasm and DCI due to higher concentration of nimodipine in cerebrospinal fluid (CSF). The risk of systemic hypotension may also be decreased with intracranial delivery. We used animal models to evaluate the feasibility of surgically implanting a silica-based nimodipine releasing implant into the subarachnoid space through a frontotemporal craniotomy. Concentrations of released nimodipine were measured from plasma samples and CSF samples. Implant degradation was followed using CT imaging. After completing the recovery period, full histological examination was performed on the brain and meninges. The *in vitro* characteristics of the implant were determined. Our results show that the biodegradable silica-based implant can be used for an intracranial drug delivery system and no major histopathological foreign body reactions were observed. CT imaging is a feasible method for determining the degradation of silica implants *in vivo*. The sustained release profiles of nimodipine in CSF were achieved. Compared to a traditional treatment, higher nimodipine CSF/plasma ratios can be obtained with the implant.

## 1. Introduction

Delayed cerebral ischemia (DCI) is a serious complication of subarachnoid hemorrhage (SAH). Nimodipine is one of few effective treatments with clinical evidence for this condition [1, 2]. Knowledge of the pathophysiology of the DCI and the underlying vasospasm has evolved in recent years to a complex entity of early brain injury, secondary injuries, and cortical spreading ischemia, instead of being pure intracranial vessel spasm [3–7]. The consequence of a diligent research for understanding the mechanism of SAH has led to the development of new tentative therapies to treat DCI, many showing promising preliminary results [8–11]. Recently, cilostazol was reported to improve outcome among SAH patients when added to a conventional treatment [12].

The severity of the complications caused by DCI and relatively ineffective current treatment strategies have led to the search of new routes to administrate nimodipine for SAH patients [13]. Intra-arterial, intrathecal, intraventricular, and intracranial routes have been described [14–18]. By obtaining higher concentrations of nimodipine in the CSF, it may be possible to reduce the risk of vasospasm [15]. Higher concentrations of nimodipine in the cerebrospinal fluid (CSF) can also increase other beneficial effects of nimodipine, for example, neuroprotection for delayed neuronal apoptosis and fibrinolysis of microthrombosis [19–22]. In an experimental SAH rat model, nimodipine inhibited cortical spreading ischemia (CSI) [22]. These alternative administration routes also help in achieving sufficient therapeutic concentrations in subarachnoid space with lower systemic nimodipine concentrations, thereby reducing the risk for systemic hypotension, which may be detrimental for patients with intracranial vasospasm.

Nimodipine in poly-D,L-lactide coglycolide (PLGA) implants has been described in the literature [23, 24]. The efficacy of intracranial nimodipine therapy is under research and the results of the studies have been encouraging [15, 25, 26]. Other calcium channel blockers incorporated into PLGA implants have been described and studied as well, including mibefradil and nicardipine [27–29]. At the moment, nimodipine is the only calcium channel blocker that is approved for treating SAH and hypothesized to have pleiotrophic effects that can improve outcome without the reduction of vasospasm [15, 19, 21, 27]. Thus that is making it an interesting candidate for developing new administration routes where intracranial administration of sustained release implant or gel seems utilitarian [30].

Calcium channel blockers formulated in a silicate matrix have not been yet described in the literature. Silica has a potential to form micropores where the active drug can bond, thus forming a sustained drug delivery system (SDDS). Silica is under active research due to its extensive scale of applications for drug delivery. Silica-based controlled drug delivery systems are competent for pure sustained-release systems but can be functionalized to response changes in pH, redox potential, temperature, biomolecules, or even magnetism and luminescence [31, 32]. Silica-based SDDS can be formulated to solid implant as well as gel with different viscosity [33]. Considering the extensive functionalizing possibilities, we formulated a silica-based sustained drug release nimodipine implant and tested its feasibility of implantation into the subarachnoid space and its local histological effects in pigs and dogs. We also measured concentrations of nimodipine in plasma and CSF and properties of the new implant and evaluated the tolerability.

## 2. Material and Methods

### 2.1. Preparation of Silica-Based Implants

**Figure 1:**
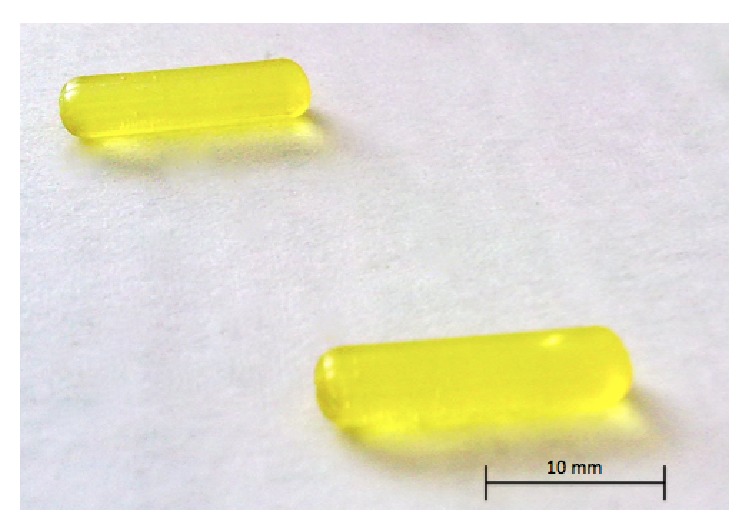
Formulated silica-based nimodipine implants, 10 wt%.

The silica implants were prepared from tetraethoxy silane (TEOS 98%, Sigma-Aldrich, USA), deionized water (Millipore, Milli-Q > 17.5 MΩ cm), ethanol (EtOH, Etax AA 99.5%, Altia, Finland), and hydrochloric acid (HCl, Merck, USA) as catalyst using the sol-gel method [34]. The molar H_2_O/TEOS ratio was 2 and the molar EtOH/TEOS ratio was 1. After hydrolysis sols were aged at 60°C temperature. After ageing of the sols, the nimodipine (or glucose) was dissolved into the sols. The pH of the sols was adjusted by sodium hydroxide solution (NaOH, Merck, USA). The final pH of the sols was 5.5. Sols were sterile filtered (Whatman, 0.22 *µ*m) and cast into polytetrafluoroethylene molds. The sols were gelled in 6 days at room temperature (RT). Formed implants were dried in a desiccator at RT. The initial concentrations of nimodipine in the sol were 10 weight (wt) % (implant A, implanted to pigs) and 15 wt% (implant B, implanted to dogs) corresponding to the amount of SiO_2_ (Figure 1).

### 2.2. Dissolution of Monoliths* In Vitro*

Degradation of silica matrices was measured in 50 mM TRIS (Trisma—preset crystals, Sigma-Aldrich) + 0.1% (wt/wt) sodium dodecyl sulfate (SDS, Merck) buffered at pH 7.4 (37°C). SiO_2_ concentrations in the dissolution medium were kept below 30 ppm to ensure* in sink* condition (free dissolution of the SiO_2_ matrix). If needed, the whole dissolution medium was changed to the fresh medium in order to keep SiO_2_ concentrations < 30 ppm. Degradation of silica matrices was also measured via flow-through dissolution method. In the flow-through dissolution method, one implant was moved into sample container with 150 mL dissolution medium and dissolution medium was changed continuously by pumping dissolution medium through sample container 347 *µ*L/min (c.a. 500 mL/day). The silica concentrations were measured with UV/VIS spectrophotometer (V-560, Jasco) analyzing the molybdenum blue complex absorbance at *λ* = 820 nm [35]. Release of nimodipine from the SiO_2_ matrices was analyzed with high pressure liquid chromatography (HPLC-UV) by CRST in Turku. The chromatographic separation was obtained on a Gemini 5 *µ* C18 110A, 150 × 2.0 mm (Phenomenex) analytical HPLC column. The mobile phase consisted of a mixture of acetonitrile and 15 mM hydrogen phosphate buffer (60 : 40 volume/volume).

### 2.3. Animal Models

Studies were performed using a domestic landrace pig and a Beagle dog model. Both groups included nine animals in total. The Beagle dogs were bred by Harlan-Winkelmann GmbH Hundezucht, Germany. The pigs were purchased from a local farm following strict health monitoring program. All procedures were made according to European Community Guidelines for the use of experimental animals and approved by Finnish National Animal Experiment Board (licenses ESAVI/2246/2011 and ESAVI/2643/2012).

Nine eight-week-old male pigs, weighing between 18,6 and 26,1 kg, and nine four-year-old male dogs, weighing 10,0–14,0 kg, were randomly selected for the operation. Board-certified neurosurgeons were blinded for the content of the implants in all surgeries. Five implants were determined to be the number of implants for testing toxicity of the nimodipine silica implant (Active 1). The group receiving one implant was considered as a regular treatment group at expected pharmacological dose and any adverse effects were not expected (Active 2). Schedule of the study and allocation to treatment groups are presented in Table 1 for the pigs and Table 2 for the dogs. Day 1 indicates the operation day.

### 2.4. Anesthesia and Analgesia

At day 1, pigs and dogs were sedated, anesthetized, intubated, and connected to a ventilator for surgery. Pigs were sedated with midazolam 1 mg/kg i.m. and xylazine 3 mg/kg i.m. and anesthesia was maintained with propofol infusion 10 mg/kg/h i.v. Dogs were sedated using dexmedetomidine 20 *µ*g/kg i.v. and anesthesia was induced with slow propofol bolus 3 mg/kg i.v. and maintained with a propofol infusion 9 mg/kg/h i.v. Heart rate, oxygen saturation, and rectal temperature were observed and recorded throughout the procedure. As a prophylactic antibiotic, cefuroxime was administered before operation, 250 mg i.v. for dogs and 750 mg i.v. for pigs. For analgesia, Fentanyl was administered to pigs intraoperatively 8–13 *µ*g/kg i.v. and 3–7 days postoperatively using a 50 *µ*g/h transdermal patch. For dogs, fentanyl was administered intraoperatively 3 *µ*g/kg i.v. and 3 days postoperatively using a 50 *µ*g/h transdermal patch.

Pigs were sedated for blood and CSF sampling with midazolam 1 mg/kg i.m. and xylazine 3 mg/kg i.m. For CSF, sampling anesthesia was induced with propofol bolus 3 mg/kg i.v. for pigs. Sedation of dogs for CSF sampling was done using dexmedetomidine 20 *µ*g/kg i.v. and a slow propofol bolus 3 mg/kg i.v. Sedation or anesthesia was not required for dogs when only venous blood samples were collected.

Pigs were sedated for CT imaging procedures with midazolam 1-2 mg/kg i.m. and xylazine 3−6 mg/kg i.m. and anesthesia was maintained with propofol 8−15 mg/kg/h i.v. infusion. For the dogs, dexmedetomidine 20 *μ*g/kg i.v. was used for the sedation and anesthesia was induced with slow propofol bolus 3−5 mg/kg i.v. and maintained as needed with propofol boluses during CT imaging.

### 2.5. Surgery

**Figure 2:**
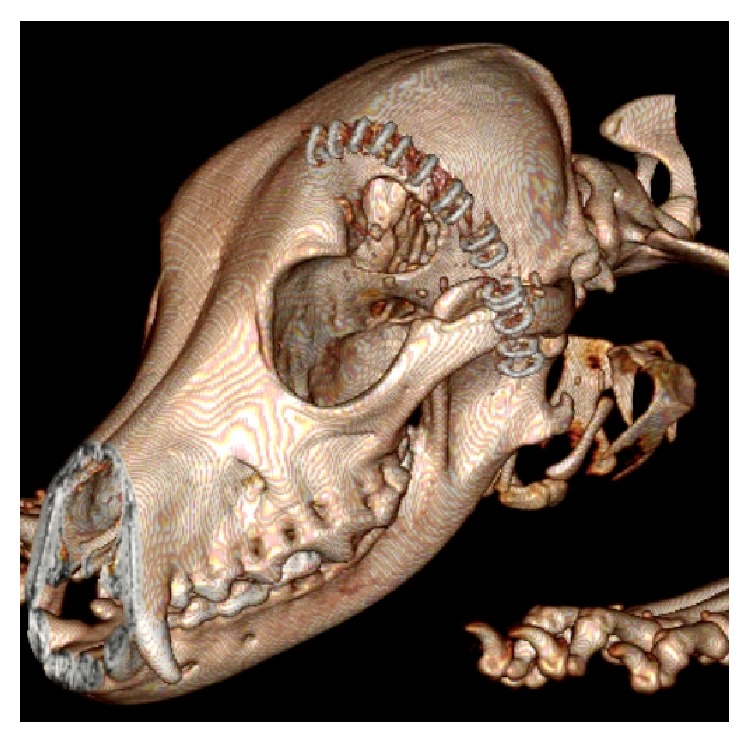
3D computed tomography reconstruction indicating the location of craniotomy and incision of the dog.

During the surgical procedures, the heart rate, the rectal temperature, and the oxygen saturation were recorded in every fifteen minutes. Similar surgical approach was used for both species. The animals were positioned in a lateral prone gesture, and a longitudinal incision was made between the left eye and ipsilateral ear. The scalp was retracted away from the zygomatic arch. The temporal muscle was cut vertically and retracted to expose the lateral frontotemporal bone at the level of the anteromedial skull base. A lateral frontotemporal craniotomy, approximately 3 cm in diameter, was made using a trephine and a craniotome in order to reach the anterior and middle skull base (Figure 2).

The dura was opened and basal cisterns were exposed under the operating microscope. In the pigs, one placebo implant, which contained 25 mg glucose, or one nimodipine implant, which contained 5 mg nimodipine, was placed towards the scull base in the basal cisterns. One placebo implant with glucose was placed into the brain parenchyma in the temporal lobe in the Placebo and active groups. Among the dogs, group Active 1 was treated with five implants containing 8.5 mg of nimodipine and group Active 2 with one implant containing 8.5 mg of nimodipine. For both pig and dog study, a sham group was included, where exactly the same procedure was performed including opening the arachnoid membrane and visualization of the optic chiasm but no implant was placed. In all groups, dura was closed using artificial graft (TachoSil, Baxter, USA, Deerfield, IL 60015-4625) and fibrin tissue glue (TISSEEL, Baxter, USA, Deerfield, IL 60015-4625). The soft tissues and wound were closed in layers. After the surgery, viability of the animals was recorded at least twice a day. During the recovery period of 7 days for pigs and 21 days for dogs, the animals were observed daily. Body weight and food and water consumption were recorded once a day. Clinical signs and rectal temperature were recorded at least twice a day.

### 2.6. Sampling for Nimodipine Concentration Measurements

Plasma and CSF samples were collected for determining nimodipine concentration. Schedules for the sampling are presented for the pigs in Table 1 and for the dogs in Table 2, respectively. Blood samples were collected from the external jugular vein into K2-EDTA tubes. In pigs, samples were collected also from ear vein. CSF samples were collected from cisterna magna through the atlantooccipital space. The samples were centrifuged (1200 G, 10 min, RT) and plasma was separated. CSF samples were centrifuged as well and supernatant was collected. Since nimodipine is very photosensitive, all samples were handled with minimal light exposure. The samples were stored frozen at −80°C nominal until transferred for analysis.

Liquid chromatography mass spectrometric (LC-MS) method was used for determination of nimodipine in dog and pig plasma and CSF samples. Sample preparation was performed by liquid-liquid extraction. The extracts were analyzed using reverse-phase chromatography followed by mass spectrometry. Lower limit of quantification (LLOQ) of the method was 0.01 ng/mL for both sample matrices.

### 2.7. Computed Tomography

The schedule for CT imaging is shown in Table 1 for pigs and Table 2 for dogs. CT studies were performed with Discovery STE PET/CT scanner (General Electric Medical Systems, Milwaukee, WI, USA) operated in a helical mode. Cerebral CT imaging was performed using an ultrafast CT protocol (335 mA, 100 kVp). Voxel size was 0,625 mm × 0,625 mm × 0,625 mm. The volume of interest (VOI) was drawn for the implant(s). A calibration phantom (13002 Model 3 CT Calibration Phantom, Mindways Software, Inc, San Francisco, CA, United States) was used prior to CT imaging and densities of the implants were calculated and corrected using the phantom data. CT image analysis was done with Carimas 2.5 software (Turku PET Centre, Turku, Finland; http://www.turkupetcentre.fi/carimas/). Density and volume of the implant were defined using cerebral CT imaging and implant degradation was evaluated. Implant degradation was calculated as a change in density and volume of the implant at different time points (for pigs: day 1 and day 7, for dogs: days 7, 14, and 21).

### 2.8. Pathologic Examination

At the end of the study period, the pigs were sedated with midazolam 1 mg/kg i.m. and xylazine 3 mg/kg i.m. and euthanized with propofol i.v. overdose and exsanguinated. Dogs were sedated with dexmedetomidine 20 *µ*g/kg i.v. and euthanized with pentobarbital i.v. overdose and exsanguinated.

The cranium of the animals was opened to expose the operation site. Meninges and brain tissue were observed for any macroscopic changes and the presence of implants was evaluated. For the histopathologic examination, samples from the meninges and brain tissue were taken from the vicinity of the implant site and from corresponding area on the opposite hemisphere. All samples were fixed in a buffered 4% formaldehyde solution. The tissue samples were embedded in paraffin, cut to a thickness of approximately 4 *μ*m, and stained with hematoxylin and eosin (HE) for histopathology. All slides were examined by a pathologist and histopathologic changes were recorded and scored with a 5-step scale (from minimal to severe).

### 2.9. Statistical Analyses

All the results are presented as means ± SD. Mixed model for repeated measurements was applied to the implant volume and density data (SAS 9.2 SAS Institute Inc., Cary, NC, USA). *P* values < 0.05 were considered as statistically significant.

## 3. Results

### 3.1. Dissolution of the Implant* In Vitro*

**Figure 3:**
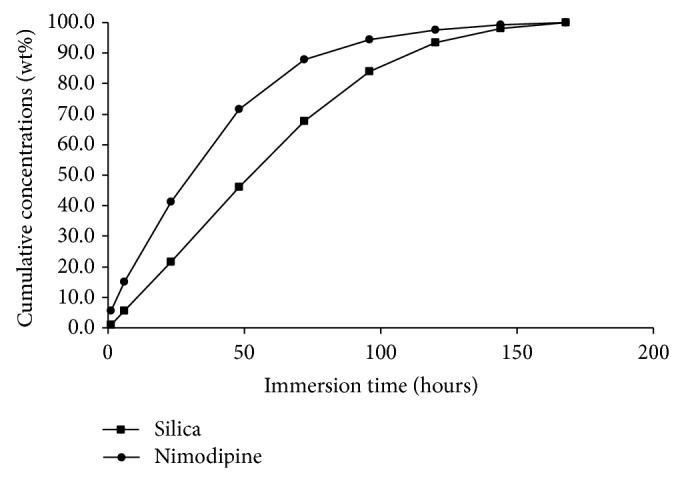
Cumulative degradation of silica (▪) and release of nimodipine (●) from implant A to dissolution medium (50 mM Tris buffer, pH = 7.4 at 37°C) in* in sink* dissolution.

**Figure 4:**
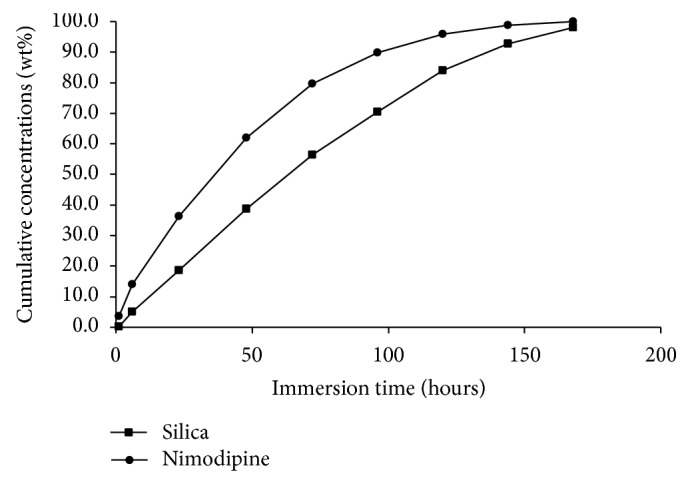
Cumulative degradation of silica (▪) and release of nimodipine (●) from implant B to dissolution medium (50 mM Tris buffer, pH = 7.4 at 37°C) in* in sink* dissolution.

**Figure 5:**
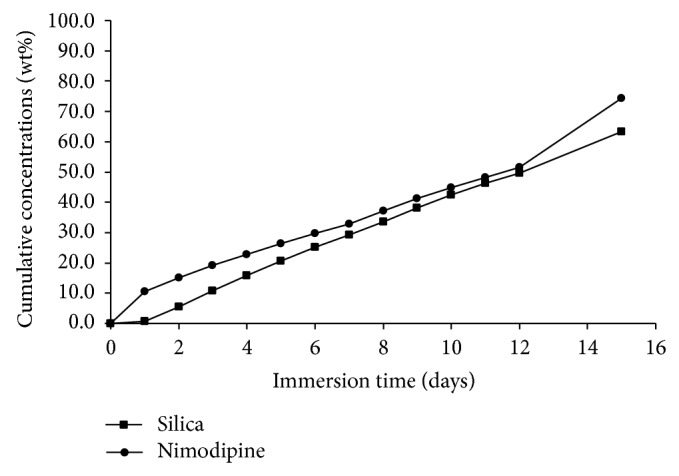
Cumulative degradation of silica (▪) and release of nimodipine (●) from implant B dissolution medium (50 mM Tris buffer, pH = 7.4 at 37°C) in flow-through dissolution. Flow rate of the dissolution medium was 500 mL/day.

Degradation rates of the silica implant formulation were measured from three parallel samples. The implant formulations were totally degraded after 3.5–4 days in* in sink* dissolution (Figures 3 and 4) and 24 days in flow-through dissolution, respectively (Figure 5). The cumulative release profiles of nimodipine were measured from the same sample solutions (pH = 7.4 at 37°C) as used for the SiO_2_ degradations measurements. Nimodipine was mainly released from the implant formulations by degradation of silica matrices as shown in Figures 3–5. The release rates of nimodipine are proportional to the SiO_2_ degradation. According to dissolution results, there were 5.0 mg and 8.5 mg of nimodipine in implant A and implant B, respectively.

### 3.2. Surgery and Recovery Period

During the surgery, heart rate, oxygen saturation, and rectal temperature were within the normal range, except for one dog (number 3), which suffered from a decrease in oxygen saturation (down to 70%). This persisted for three minutes at the end of the operation due to a malfunction of the ventilator. Postoperatively signs of systemic infection were not manifested and rectal temperatures were within a normal range throughout the recovery period. All clinical findings of the dogs during the 21-day recovery period are presented in Table 3. During the 7-day recovery period in pigs, no clinical signs of morbidity or changed behavior were noted. Body weights of the dogs were slightly decreased in each group: on average, in group Active 1, a decrease of 7% (*n* = 2) was seen, in Active 2, a decrease of 4% was seen, and, in the sham group, a decrease of 2% was noted. Food consumption in all dog groups remained normal during the 21-day recovery period. In pigs, body weight gain was 7%–18% and can be considered normal in each group, respectively.

Mortality of this nonclinical experiment was one animal. Animal number 3 (dog) developed refractory complex partial epilepsy and had to be euthanized on day 3. Another dog from the same group (number 2) had epileptic seizures on day 4 and was treated with phenobarbital medication. The dog responded well to the treatment. Postoperative eye infections were treated with local ophthalmic ointment containing fusidic acid and the infections responded well to the treatment.

### 3.3. Pathological Examination

In the macroscopic examination of the pigs, no abnormalities were detected at the implantation area. In dogs, local attachment of meninges to the implant site was seen, which prevented the evaluation of the actual implants. This lesion was not seen in the sham group.

Summary of the histopathological examination is shown in Table 4. Dog number 3 (Active 1) was sacrificed on day 3 and therefore excluded from these results. In histopathological examination of the operation site, local inflammatory reaction characterized by infiltration of mononuclear and granulocytic cells was seen in the meninges of both species. In pigs, there was an eosinophilic component whereas, in dogs, the inflammation was more neutrophilic. Multinucleated giant cells indicating foreign body reaction and foreign implant material were seen mainly in dogs receiving five implants. Additional findings were mild to moderate fibrosis, vascular regeneration, and mild hemorrhage on the meninges in all groups. In general, the changes were more frequent and severe in the dogs, but no clear differences in the severity of changes could be seen between sham groups and implanted groups. Furthermore, there were no histopathological differences between the two implant types (glucose versus nimodipine as active ingredient). In all groups, the underlying brain parenchyma was only mildly affected by perivascular mononuclear cuffing or spreading of inflammatory infiltrate from the meninges. In the dog sham group and in the Active 1 group, one animal in each group showed focal degeneration and gliosis in the deeper parenchyma with no direct connection to implant site. No significant abnormalities were observed in the samples from opposite hemisphere.

The implant site located in the brain parenchyma was present in the samples from two pigs (numbers 23D and 23F). The local changes around the parenchymal implant site did not show major signs of foreign body reactions. There was local inflammatory reaction with foamy macrophages and minimal to marked degeneration in the surrounding parenchyma, but not more than that could be expected from the procedure itself and intraparenchymal implantation.

### 3.4. Nimodipine Concentrations

In pigs, calculated CSF/peripheral venous plasma ratio was 1.31 ± 1.34 on day 5 and 0.886 ± 0.255 on day 7. Correspondingly in dogs receiving active implant, after one hour in 5 × 8,5 mg nimodipine group, the CSF/jugular plasma ratio was 0.002 ± 0.0005 and in 8,5 mg nimodipine group 0.149 ± 0.174. The sustained release profile of nimodipine in CSF was achieved for 21 days in dogs and 7 days in pigs (Table 5).

### 3.5. Computed Tomography Imaging

**Figure 6:**
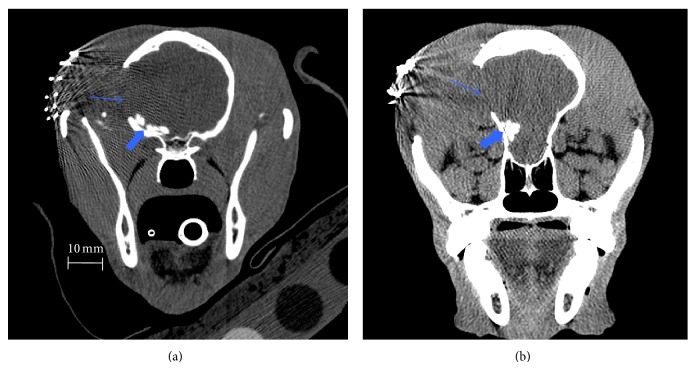
Coronal CT scanning of the dogs number 1 (A) and number 2 (B) one week postoperatively. (a) Five implants in the basal cisterns (thick arrow), no clinical signs of intolerance observed. Craniotomy site is indicated by thin arrow. (b) Scanning indicates a too rostral positioning of the implants, and five implants had formed pile-like formation protruding into the parenchyma of the frontal lobe (thick arrow). Craniotomy site is indicated by thin arrow.

Clinical analyses of the CT data showed the location and degradation of the implants in the subarachnoid space in pigs and dogs. The implants were correctly placed at the base of the cranium in the Sylvian fissure in the subarachnoid space in all pigs. This was also noted in most dogs, except for dogs number 2 and number 3 in group Active 1 (Figure 6). Dog number 3 had to be euthanized at day 3 and CT imaging was therefore not performed. CT imaging of dog number 2 showed a too rostral positioning of the implants, and five implants had formed pile-like formation protruding 6,5 mm into the parenchyma.

**Figure 7:**
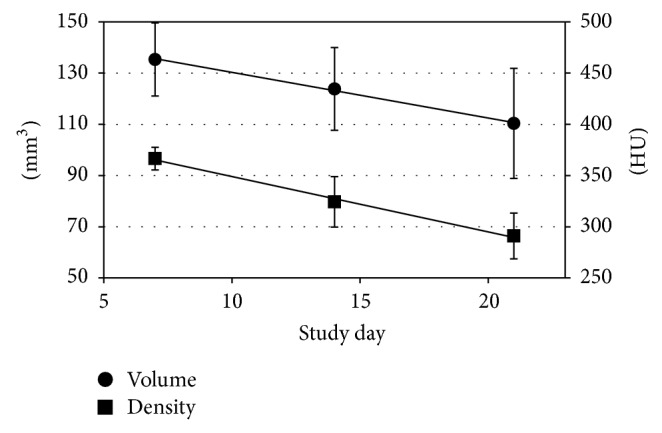
Implant volume and density curves for dogs. HU = Hounsfield Unit.

In dogs, implant size and density were decreased in implants measured between weeks 1 and 3 (Figure 7). Degradation was very linear. Based on slopes of the degradation curves, density was decreased faster than volume (Table 6).

## 4. Discussion

The objective to deliver nimodipine biodegradable silica-based implants into the subarachnoid space through frontotemporal craniotomy in pigs and dogs was met. The surgery and the recovery period of the pigs were passed without any complications. In the dogs, two animals developed epileptic seizures, both receiving five 8,5 mg nimodipine implants into the basal cisterns. One had to be euthanized due to refractory epileptic seizures, the second responded well to the phenobarbital treatment, and the medication could be terminated by day 20. Refractory epilepsy of the dog number 3 was probably caused by the combination of hypoxia during surgery and mild protrusion of the implants into the parenchyma that was observed in autopsy. In the CT imaging, dog number 2 showed improper placement of implants and protrusion of the implants into parenchyma, which may have caused the seizures. This dog showed also focal parenchymal degeneration in the histopathology, which may have been related to the protrusion of implants. Although the evidence is suggesting direct compression of the frontal lobe parenchyma to be behind the seizures, in theory, it is possible that the etiology may rise from the surgery itself or as a direct adverse effect of the implants.

In the beginning of the study, five implants were determined to be the number of implants for testing toxicity levels of the nimodipine silica-implant. This number of implants was also the maximum that could be delivered into the basal cisterns of a beagle dog. In histopathological examination of dogs and pigs, no major differences were noted between groups regardless of the presence of implants and most of the findings were present also in the sham-operated animals, indicating that these findings were related to the procedure itself and that the implants are locally tolerable. Foreign body type reaction was mainly seen in dogs receiving five implants, which can be directly related to the larger amount of implant material. Additionally, the implantation directly into the brain parenchyma of pigs did not produce widespread tissue damage around the implant. Therefore silica-based biodegradable materials can be considered for use in the release of the therapeutic agents for an intracranial treatment of vasospasm and DCI.

Based on the nimodipine concentrations in the CSF, sustained release profile of nimodipine was achieved for 21 days in dogs and 7 days in pigs. Interestingly, despite the intracisternal location of the implants, the increase of nimodipine concentration in CSF was modest. The samples were taken through atlantooccipital space from cisterna magna and therefore the low concentrations could be due to a net flow of CSF towards arachnoid villi and sinuses, resulting in a lower concentration in CSF located extracranially or cisterna magna. There might be some specific features in implant or nimodipine amount that leads to lower CSF concentrations. For example, Cook et al. reported significantly lower nimodipine concentration in CSF when using 30 mg loaded nimodipine PLGA microparticles compared to the 10 mg loaded PLGA microparticles [15]. Indeed, the SAH and also the neurosurgical procedure itself may compromise the normal CSF dynamics. Sufficient CSF dynamics are required for the implant dissolution and distribution of nimodipine. Thus pharmacokinetics and the degradation of the implants after SAH need to be studied in further trials.

The physical degradation of the silica implant and prediction of the implant behavior* in vivo* were feasible to follow using CT imaging. High dissolution rates in the basal cisterns may be induced by increased circulation of spinal fluid. A rapid decrease of the density is in line with the physical properties of the implant matrix. The degradation of silica matrix occurs constantly throughout the implant; therefore, the implant size decreases slower than its density. Based on the results obtained in this study, CT imaging can be a feasible method for determination of the degradation of the silica implants* in vivo*. More studies are needed to clarify if CT imaging can be used to estimate the drug release profile of silica implants.

## 5. Conclusions

In conclusion, we found that a biodegradable silica-based implant can be used as a material for an intracranial drug delivery system and no major histopathological foreign body reactions were observed. A sustained release profile of the nimodipine implant was achieved and compared to the traditional treatment; higher nimodipine CSF/Plasma ratios may be obtained. CT imaging proved to be a feasible method for observing the* in vivo* degradation properties of a silica-based implant. More studies are needed to understand the pharmacokinetics of the intracranial nimodipine delivery systems, and further formulation development of the implant to a sustained drug release gel may be of interest from a clinical point of view.

## Figures and Tables

**Table 1:** Allocation of treatment groups and schedule of the pig study.

Group	Procedure	Parenchyma implant	Animals per group	Animal IDs
Sham	Sham operation	No implant	3	22A, 22B, and 23C
Placebo	Placebo implant 25 mg glucose × 1	Placebo implant	3	22C, 23A, and 23F
Active	Nimodipine implant 5 mg × 1	Placebo implant	3	23B, 23D, and 23E

Samples	Day 1	Day 4	Day 5^1^	Day 7

Plasma (ear vein)			x	x
Plasma (vena jugularis)	x	x	x	x
Cerebrospinal fluid			x	x
CT	x			x^2^

^1^Day 6 on animals 22A, 22B, and 22C.

^2^Only animals 23A and 23D underwent CT-imaging.

**Table 2:** Allocation of treatment groups and schedule of the dog study.

Group	Procedure	Parenchyma implant	Animals per group	Animal IDs	
Active 1	Nimodipine implant 8.5 mg × 5 (total 42.5 mg)	NA	3	1, 2, 3	
Active 2	Nimodipine implant 8.5 mg × 1	NA	3	4, 5, 6	
Sham	Sham operation	NA	3	7, 8, 9	

Samples	Day 1	Day 5	Day 7	Day 14	Day 21

Plasma (vena jugularis)	1 h, 1.5 h, 2.5 h, 4 h, 6 h, and 22 h (on day 2)	x	x	x	x
Cerebrospinal fluid	1 h		x	x	x
CT			x	x	x

NA = not applicable.

**Table 3:** Clinical signs of the dogs during the 21-day recovery period.

Group	Dog number	Epileptic seizure	Other/notice
Active 1	1	No	—
	2	Yes	Epileptiformic seizure on day 4. Treated with phenobarbital medication 2.5 mg/kg twice a day on days 4–12. The medication was reduced to 2.5 mg/kg once a day on day 13 and ended on day 20.
	3	Yes	Epileptiformic seizure 6 hours postoperatively. Epileptiformic seizures were refractory to phenobarbital medication. The animal had to be euthanized on day 3 according to the protocol. It was not replaced.

Active 2	4	No	24 h postoperatively, conjunctivitis.
	5	No	24 h postoperatively, vomiting.
	6	No	24 h postoperatively, conjunctivitis.

Sham	7	No	—
	8	No	24 h postoperatively, conjunctivitis.
	9	No	—

**Table 4:** Summary of histopathological findings and severity in pigs and dogs in each group. The number of affected animals in each group is shown. Severity score indicates the recorded minimum and maximum severity in each group. Only one score is shown if all animals had same severity or only one animal was affected. *N* = 3 in each group.

Lesion	Pigs			Dogs		
	Sham	Placebo	Active	Sham	Active 1^*^	Active 2
*Infiltration of mononuclear cells (meninges) *	1	3	3	3	2	3
Severity score, min/max^∧^	*++++ *	*++/+++ *	*+/++++ *	*++++ *	*++++ *	*+++/++++ *
*Infiltration of granulocytes (meninges) *	1	1	1	3	2	3
Severity score, min/max	++++	+	++	+/+++	+++	+/++
*Multinucleated giant cells and foreign material (meninges) *	0	0	0	0	2	1
Severity score, min/max	—	—	—	—	++	++
*Fibrosis (meninges) *	1	3	1	3	2	3
Severity score, min/max	++	+	++	++/+++	+++/++++	++/++++
*Vascular regeneration (meninges) *	0	2	0	3	1	3
Severity score, min/max	—	+++	—	++	++	++
*Hemorrhage +/− hemosiderophages (meninges) *	1	2	1	2	1	3
Severity score, min/max	+	+/+++	++++	++	++	++
*Perivascular cuffing (parenchyma) *	0	0	1	3	2	3
Severity score, min/max	—	—	+	+/+++	++	+/++

^*^In this group, *n* = 2. Animal number 3 was excluded due to preterminal sacrifice.

^∧^+: minimal, ++: slight, +++: moderate, ++++: marked, and —: not present.

**Table 5:** Concentrations of nimodipine at different time points after surgery in dogs and pigs (ng/mL).

	1 h		Day 7		Day 14		Day 21	
	Jugularis	CSF	Jugularis	CSF	Jugularis	CSF	Jugularis	CSF
Nimodipine 8.5 mg implant in dogs	17.79 ± 15.17	2.19 ± 1.90	6.13 ± 4.66	0.025 ± 0.009	11.51 ± 3.77	0.030 ± 0.008	3.05 ± 1.53	0.013 ± 0.004
Nimodipine 5 × 8.5 mg implant in dogs	63.9 ± 18.7	0.110 ± 0.005	37.8 ± 15.8	0.095 ± 0.008	36.5 ± 9.97	0.083 ± 0.009	32.9 ± 8.77	0.034 ± 0.004

	Day 5				Day 7			
	Jugularis		Ear	CSF	Jugularis	Ear		CSF

Nimodipine 5 mg implant in pigs	0.045 ± 0.008		0.027 ± 0.005	0.027 ± 0.022	0.054 ± 0.006	0.045 ± 0.020		0.038 ± 0.017

**Table 6:** Statistical parameters of implant volume and density data for dogs. Implant density is decreasing faster based on slope of the degradation curves.

	Intercept	Slope	*P* value
Volume	148.12	−1.7829	0.0442
Density	402.93	−5.4000	0.0002

## Abbreviations

CRST: Clinical research services Turku
CSF: Cerebrospinal fluid
CSI: Cortical spreading ischemia
CT: Computed tomography
DCI: Delayed cerebral ischemia
LC-MS: Liquid chromatography-mass spectrometry
PET: Positron emission tomography
PLGA: Poly D,L-lactide coglycolide
SAH: Subarachnoid hemorrhage
SDDS: Sustained drug delivery system
Silica: SiO_2_.
